# A Complex Cardiac Mass Originating from Interatrial Septum in a Patient with History of Kidney Cancer: A Case Report and Literature Review

**DOI:** 10.1155/2018/1764057

**Published:** 2018-05-31

**Authors:** Negar Faramarzi, Sumit Sohal, Roshanak Habibi, Muhammad Sikander Akbar

**Affiliations:** ^1^Department of Medicine, Presence Saint Francis Hospital, 355 Ridge Avenue, Evanston, IL 60202, USA; ^2^Department of Cardiology, Presence Saint Francis Hospital, 355 Ridge Avenue, Evanston, IL 60202, USA

## Abstract

Cardiac tumors are a rare phenomenon, and most cases are secondary to metastatic cancers rather than primary tumors. Renal cell carcinoma (RCC) is notorious for metastasis to cardiac tissue. Tumor thrombus migration to the renal vein and inferior vena cava happens in up to 10% of patients with RCC. Transitional cell carcinomas are another form of renal cancer, which may metastasize to the heart and are associated with widespread metastasis. Here, we report a patient with a past medical history of metastatic transitional cell cancer of renal pelvis under treatment with checkpoint inhibitor therapy presenting with shortness of breath. He had disseminated metastasis to bones, pleural space, lungs, and muscles. A large mass was found in the interatrial septum with invasion to the right and left atrium. The mass had a cystic component protruding into the left atrium. He passed away a few days after presentation.

## 1. Introduction

The estimated incidence of primary cardiac masses is less than 0.1 percent, while secondary cardiac masses are 20–40 times more common [1]. Renal cell carcinoma (RCC) is one of the common causes of secondary cardiac tumors, and several cases of late metastasis from RCC have been reported years after curative excision of the primary tumor [2, 3]. Transitional cell carcinoma accounts for nearly 10 percent of all renal cancers, causing 3800 cases in the United States annually [4]. Cardiac involvement can occur in cases of widely metastatic transitional cell carcinoma [5, 6].

Cardiac masses in renal cancers can be asymptomatic or can present with life-threatening medical emergencies, such as tamponade or outflow obstruction, causing hypotension, tachycardia, and shortness of breath. Cardiac involvement, especially through the lymphatic system, has been associated with widely metastatic disease [7]. In patients with a pertinent history of renal cancer, echocardiography can help to exclude cardiac masses as a cause of these symptoms [1, 8].

## 2. Case Report

A 32-year-old male presented with progressive shortness of breath and leg swelling. He had a past medical history significant for HIV disease and metastatic transitional cell cancer of the renal pelvis and left nephrectomy. He had metastatic involvement of the spinal bones, mediastinal lymph nodes, and lungs. He received gemcitabine and cisplatin followed by salvage treatment with atezolizumab. He was also on combination antiretroviral therapy (elvitegravir/cobicistat/emtricitabine/tenofovir/alafenamide) with undetectable viral load and CD4 count of 444 cells/mm^3^.

**Figure 1:**
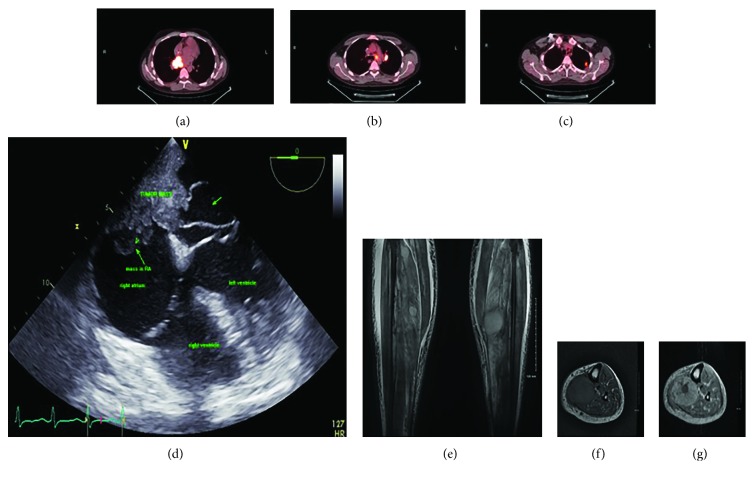
(a–c) PET scan images demonstrate increased uptake within nodes in the aortic pulmonary window, left hilum, and a mass in the left upper lung. (d) Transesophageal echocardiography shows large cardiac mass invaded to the intra-atrial septum and right and left atrium with cystic lesion protruding to the left atrium. (e–g) MRI with and without contrast shows multiple enhancing neoplastic masses in the muscle compartment in both lower extremities which are believed to be due to metastatic lesions.

The physical exam was remarkable for tachycardia, respiratory distress, decreased breath sounds in the right hemithorax, and lower extremity swelling. A chest X-ray showed a right-sided pleural effusion and an enlarged nodular density in the left upper lobe. Laboratory data revealed anemia and an elevated troponin I. Patient symptoms resolved partially after he received a blood transfusion and underwent therapeutic thoracentesis. Transthoracic echocardiography revealed normal left ventricular function and a large, mobile, cystic mass in the right and left atrium. Transesophageal echocardiography revealed a large mass composed of solid and cystic components. The solid component, a 5 × 2.3 cm^2^ mass, had invaded the basal half of the interatrial septum and the cystic component was found to protrude into the left atrium (Figure 1(d)). Multiple enhancing neoplastic masses in the muscular compartment in both calves were detected on MRI of the lower extremities, which were in favor of neoplastic lesions (Figures 1(e)–1(g)). The patient was not a candidate for surgery considering the extent of disease and he expired few days after presentation.

## 3. Discussion

In 9% of patients who died of cancer, cardiac metastases have been found at autopsy. Malignant melanomas, lung cancer, breast cancer, and renal carcinoma are commonly associated with secondary cardiac tumors. Cardiac involvement should be considered as differential diagnosis whenever a patient with known malignancy develops cardiovascular symptoms or signs, arrhythmia, or ECG changes [9].

Cardiac metastasis of transitional cell carcinoma has been previously reported in the right ventricle, left atrium, and pericardium. Malde et al. reported a case of papillary muscle rupture following myocardial infarct secondary to tumor emboli [10]. The mechanism of spread is hypothesized to be either hematogenous, with macro- or micro-dissemination through the IVC typically involving the endocardium and cavity, or through the lymphatic system, which involves the myocardium and pericardium. The latter is more commonly associated with disseminated disease progression. There are several reports in the literature of endocardial metastasis, which could be the case in our patient who had disease of the interatrial septum and evidence of mediastinal lymph node involvement 2 months prior to presentation (Figures 1(a)–1(c)) [1, 7].

Histology of the mass was unavailable; therefore, other differential diagnoses such as a second primary malignancy cannot be ruled out. However, the metastatic origin of the mass is more likely, considering widespread metastatic disease and the results of a prior biopsy of a pulmonary nodule, which revealed high-grade transitional cell carcinoma.

Five-year cancer-specific survival rate of patients with high-grade transitional cell carcinoma is 35% [11]. The chemotherapy regimen of MVAC (methotrexate, vinblastine, doxorubicin, cisplatin) or GC (gemcitabine, cisplatin) is recommended for patients with advanced disease [12, 13]. Checkpoint inhibitor immunotherapy can be used for patients with progressive disease following platinum-based chemotherapy [14, 15].

Management of secondary cardiac mass is a therapeutic challenge. Surgical resection can play an important role in palliation of isolated cardiac metastasis. Sobczyk et al. reported a case of transitional cell carcinoma metastasized to the left atrium, which was successfully removed by surgery; however, the patient passed away one month later because of progression of neoplastic disease [16]. Unfortunately, our patient was not a candidate for surgery because of his widespread neoplastic disease, and he died of respiratory failure a few days after the presentation.

## Supplementary Materials

Supplementary Materials Transesophageal echocardiography demonstrated a large mass composed of solid component in interatrial septum and cystic part protruding in the left atrium.
